# Evidence that polymorphonuclear neutrophils infiltrate into the developing corpus luteum and promote angiogenesis with interleukin-8 in the cow

**DOI:** 10.1186/1477-7827-9-79

**Published:** 2011-06-08

**Authors:** Sineenard Jiemtaweeboon, Koumei Shirasuna, Akane Nitta, Ayumi Kobayashi, Hans-Joachim Schuberth, Takashi Shimizu, Akio Miyamoto

**Affiliations:** 1 Graduate School of Animal and Food Hygiene, Obihiro University of Agriculture and Veterinary Medicine, Obihiro 080-8555, Japan; 2 Institute of Immunology, University of Veterinary Medicine Hannover, 30173 Hannover, Germany

## Abstract

**Background:**

After ovulation in the cow, the corpus luteum (CL) rapidly develops within a few days with angiogenesis and progesterone production. CL formation resembles an inflammatory response due to the influx of immune cells. Neutrophils play a role in host defense and inflammation, and secrete chemoattractants to stimulate angiogenesis. We therefore hypothesized that neutrophils infiltrate in the developing CL from just after ovulation and may play a role in angiogenesis of the CL.

**Methods and Results:**

Polymorphonuclear neutrophils (PMN) were detected in CL tissue by Pas-staining, and interleukin-8 (IL-8, a neutrophil-specific chemoattractant) was measured in supernatant of the CL tissue culture: considerable amounts of PMNs and the high level of IL-8 were observed during the early luteal phase (days 1-4 of the estrous cycle). PMNs and IL-8 were low levels in the mid and late luteal phases, but IL-8 was increased during luteal regression. The PMN migration in vitro was stimulated by the supernatant from the early CL but not from the mid CL, and this activity was inhibited by neutralizing with an anti-IL-8 antibody, indicating the major role of IL-8 in inducing active PMN migration in the early CL. Moreover, IL-8 stimulated proliferation of CL-derived endothelial cells (LECs), and both the supernatant of activated PMNs and IL-8 stimulated formation of capillary-like structures of LECs.

**Conclusion:**

PMNs migrate into the early CL partially due to its major chemoattractant IL-8 produced at high levels in the CL, and PMNs is a potential regulator of angiogenesis together with IL-8 in developing CL in the cow.

## Background

The corpus luteum (CL) is a unique endocrine organ that develops from the ovulated follicle during the sexual cycle. The main function of the CL is to secrete a large amount of progesterone (P), which is essential for establishment and maintenance of pregnancy. After ovulation, inadequate function of the CL is one of major causes of infertility in cows [1]. Angiogenesis is fundamental to the normal development of the CL in many species. One of the major angiogenic factors, basic fibroblast growth factor (FGF2), is generally involved in cell growth, differentiation, transformation and angiogenesis. Gospodarowizc et al. [2] have been shown that FGF2 is produced in the bovine CL and stimulates neovascularization and proliferation of a wide variety of cells, such as vascular smooth muscle cells, granulosa cells and endothelial cells. Additionally, vascular endothelial growth factor A (VEGFA, mainly localized in the cytoplasm of luteal cells) also is major player for angiogenesis and plays a fundamental role in maintenance of the vasculature in the CL when it is no longer undergoing active angiogenesis [3].

Cytokines and chemokines, such as prostaglandins (PGs) [4], tumor necrosis factor α (TNFα) [5], interleukin (IL)-1 [6] and IL-8 [7,8] are found in high concentrations in the follicular fluid in the pre-ovulatory phase. Consequently, high numbers of neutrophils and macrophages infiltrate in the pre-ovulatory follicle at the time of ovulation [9]. Indeed, neutrophil depletion by administration of a monoclonal antibody against neutrophils reduced the ovulation rate in rats, indicating a critical role for neutrophils in ovulation [10]. After ovulation, a multitude of leukocytes, such as macrophages and eosinophils are located in the luteinizing theca area in the developing CL [11-13]. This series of phenomena from ovulation to luteal development involves bleeding, immune cell infiltration, tissue remodeling and angiogenesis, implying that the development of the CL following ovulation is a kind of physiological injury with an inflammatory response. In general, polymorphonuclear neutrophils (PMN) are the first cells recruited to inflammatory sites, providing cytokines and proteolytic enzymes. Moreover, PMNs secrete VEGFA [14] and induce the sprouting of capillary-like structures of endothelial cells *in vitro *[15], suggesting that PMNs have a potential role, not only in phagocytosis, but also in the regulation of angiogenesis.

IL-8 is a small protein (8.4 kDa) and produced by macrophages, endothelial cells and neutrophils [15,16]. IL-8 has been shown to be a neutrophil-specific chemoattractant [16], and IL-8 is involved in angiogenesis, cell proliferation, and apoptosis [16,17]. In the ovary, IL-8 is detected in theca, granulosa, granulosa-lutein, and vascular endothelial cells in humans [18,19] and rabbits [20]. Goto *et al*, [21] observed that IL-8 injection increased follicular growth and capillary vessel densities around the follicles in rats. Furthermore, treatment of anti-IL-8 antibody inhibited the hCG-induced ovulation rate [22], suggesting that IL-8 participates in the regulation of ovarian function to induce ovulation. However, no evidence has been shown regarding the role of neutrophils and IL-8 in the life span of the bovine CL.

We hypothesized that PMNs infiltrate in the developing CL from just after ovulation and may play a role in angiogenesis of the CL. Therefore, in the present study, we investigated the localization of PMNs and their chemoattractant, IL-8, in the bovine CL during the estrous cycle and effects of PMNs and IL-8 on the function of luteal endothelial cells *in vitro*.

## Methods

### Experiment 1: the estrous cycle

Ovaries were collected from a local slaughterhouse and CLs were classified according to stage of the estrous cycle by macroscopic observation (color and size of the CL) and weigh of the CL as described previously [23]. The stages of the estrous cycle were estimated as follows: early, (i) days 1-2, (ii) days 3-4, (iii) days 5-7; mid, days 8-12; late, days 13-16; and luteal regression, days 18 or greater of the estrous cycle (n = 4-5 in each stage). To use immunohistochemical analysis, CL samples were fixed with 10% formaldehyde for 24 h, and embedded in paraffin wax according to the standard of histological technique. In addition, CL was minced and approximately 0.1 g of luteal tissues was placed into a 1.5-ml microcentrifuge tube with 400 μl of TRIzol reagent (Gibco BRL, Gaithersburg, MD) and stored at -80°C for further analyzed. Also, to detect IL-8 concentration from the CL, the early- (days 3-4), mid-, late and regressing phase of CLs were cut into cubes (length, 4 mm; side, 2 mm; thickness, 2 mm; n = 4-5 in each stage). The CL tissues were washed in DMEM/F-12 (Invitrogen Corporation, Tokyo, Japan) with 0.1% fetal bovine serum (FBS; Invitrogen Corporation) for 15 min. Subsequently, the CL tissues were rinsed with phosphate buffered saline (PBS) to eliminate the effect of the FBS and transferred to serum-free DMEM/F-12 medium medium containing 0.1% BSA, 0.1% gentamicin solution (50 mg/L, SIGMA, St. Louis, MO, USA), and 1% amphotericin B solution (2.5 mg/L, SIGMA). CL tissue was set in a 48-well plate (1 tissue/well). Incubation was carried out for 8 h at 37°C. Then, supernatant of the CL tissue was harvested, and IL-8 within the supernatant was measured using an ELISA kit for bovine IL-8 (USCN Life Science Inc., Wuhan, China). The standard curve for IL-8 ranged from 15.6 to 1000 pg/ml.

#### Luteal cells (LCs) and luteal endothelial cells (LECs) culture

The CLs of the mid luteal phase were collected at local slaughterhouse, and dispersed using collagenase IV (SIGMA). The luteal stages were classified as mid (days 8-12 of the estrous cycle) by macroscopic observation of the ovary as described previously [24]. LCs was isolated from the bovine mid CL (Days 8-12 of the estrous cycle) using magnetic tosylactivated beads coating with BS-1 lectin (binds glycoproteins on the bovine endothelial cells), BS-1 negative cells were assessed as enriched LCs (including smooth muscle cells and pericytes) as described previously [25]. Before experiment, we confirmed the mRNA expression of steroidogenic acute regulatory protein and endothelial nitric oxide synthase as a maker of LCs in BS-1 negative cells and a maker of LECs in BS-1 positive cells, respectively (data not shown). LCs were cultured in DMEM/F-12 medium containing 5% FBS, 2.2% NaHCO3, gentamicin solution, and amphotericin B solution. Cytokeratin- negative LECs isolated from the CLs of the cows during the mid-luteal phase were used as described previously [26]. LECs were grown on plates pre-coated with 1% Vitrogen in DMEM/F-12 medium containing 5% FBS, 2.2% NaHCO3, gentamicin, and amphotericin B solution. All experiments in the present study were carried out on LECs from passages 5 to 8. For analysis of mRNA expression, LCs and LECs were cultured for 24 h at 37°C, then placed into a 1.5-mL microcentrifuge tube with 400 μL of TRIzol reagent and stored at -80°C until analysis.

### Experiment 2: *PMN *migration capacity of early- and mid-phase luteal tissue

#### PMN isolation

Whole blood (20 ml) was mixed with an equal volume of PBS. The suspension was layered onto Ficoll-Paque solution (Lymphoprep, AXIS-SHIELD, Norway) and then centrifuged at 1000 × *g *at 10°C for 30 min as described previous study [27,28]. The plasma and buffy coat (population of mononuclear cells) were discarded. Contaminating red blood cells were washed in hypotonic distilled water for approximately 10 sec. Isotonicity was restored by the addition of twice concentrated-PBS. PMNs were centrifuged at 500 × *g *at 10°C for 10 min and then the cell pellet was washed twice with PBS. Isolated PMNs were resuspended at a concentration of 2 × 10^6 ^cells/ml in RPMI 1640 (Invitrogen) with 0.1% FBS. PMNs viability was 99% as assessed by the Trypan blue staining. To check the purity of PMNs before using for the experiment, the purity of PMNs was > 95% and these cells resulted in nearly pure granulocyte populations as determined by flow cytometric evaluation (Beckman Coulter, Inc., CA, UAS) [27,28]. Additionally, we observed giemsa-stained PMNs by microscope, these cells were clear granule and segmented nuclear. Although peripheral granulocytes include neutrophils, eosinophils and basophils in generally, the purity of neutrophils (2-5 lobes of nuclear and finely-granular) was > 95% in these PMNs by microscope observation in the present study since character was different between neutrophils and eosinophils (double nuclear and coarsely-granular).

#### Migration assay by culture medium and detection of IL-8

Supernatants of the early- (days 3-4) and mid- (days 8-12) phase CLs were used (n = 4 in each stage) as described above. PMN chemotaxis was evaluated using a 10-well microchemotaxis chamber (Neuro Probe, Inc., Gaithersburg, MD, USA). Test solutions in the bottom chamber were separated from leukocytes in the upper chamber by an 8- μm pore-sized filter (Neuro Probe, Inc.). The following solutions (300 μL) were pipetted into the bottom chamber: (i) medium alone, (ii) supernatant derived from the early- or mid-phase CL tissue culture, and (iii) recombinant bovine IL-8 (50 ng/ml) as a positive control. After instrument assembly, PMNs were added to the upper chamber (250 μl/well; 2 × 10^6 ^cells/ml). After a 3 h incubation at 37°C in 5% CO_2_, migrated cells in the bottom chamber were counted using a light microscope.

#### Inhibition of IL-8 function in PMN migration in early-phase CL supernatan

To investigate the possible role of IL-8 as a neutrophil-specific chemoattractant in early-phase CL tissue culture supernatant, we inhibited IL-8 function using anti-IL-8 monoclonal antibody reacted with bovine IL-8 (Ab; MAB1044, Millipore Corporation, Australia) and then performed a transmigration assay. The supernatant of each CL was incubated with anti-IL-8 Ab (30 μg/ml) or same dose of normal IgG as control at 37°C for 1 h in duplicates (n = 4 in each group). Following incubation, media were analyzed for PMN migration as described above. The following solutions (300 μl) were pipetted to the bottom chamber: (i) medium alone, (ii) supernatant derived from the early CL culture with or without IL-8 Ab pre-treatment, and (iii) recombinant IL-8 (50 ng/ml) as a positive control. After instrument assembly, PMNs were added to the upper chamber (250 μl/well; 2 × 10^6 ^cells/ml). After incubation at 37°C in 5% CO_2 _for 3 h, migrated cells in the bottom chamber were counted using a light microscope.

### Experiment 3: effect of PMNs and IL-8 on live cell number of luteal endothelial cells and capillary tube formation

#### Live cell number of luteal endothelial cells

LECs as described above was used in the experiment. These cells were grown in DMEM/F-12 containing 5% FBS (1 × 10^5 ^cells/well in 24-well plates) for 24 h at 37°C. Then, LECs were rinsed with PBS twice, and fresh medium was replaced with medium containing IL-8 (1, 10 and 100 ng/ml) for 48 h in DMEM/F-12 medium containing 0.1% FBS. Then, cells were trypsinization and the number of LECs was counted by light microscope as live cell number of LEC without dead cells staining with Trypan blue.

#### Capillary tube formation on matrigel of luteal endothelial cells

Capillary-like tube formation of LECs was evaluated as previously described [29]. Briefly, 48-well plate was coated with 200 μl/well of BD matrigelTM basement membrane (BD biosciences, Bedford, MA, USA) at 4°C, which was then allowed to polymerize at 37°C for at least 1 h. LECs (2 × 10^4 ^cells/well) were cultured in a final volume of 0.5 ml in LEC culture medium containing IL-8 (1, 10 and 100 ng/ml) or the supernatants from PMNs (n = 4 in each group). To obtain the supernatant from PMNs, PMNs from the early luteal phase were cultured in 48-well plates (1 × 10^6 ^cells/well) in the presence of N-formyl-methionyl-leucyl-phenylalanine (fMLP, 10 and 100 nM) or without fMLP as control for 1 h at 37°C as previously described [15]. After stimulation of PMNs with fMLP, supernatant of activated PMNs were harvested and used for the assessment of capillary tube formation (n = 4 in each group). After 8 h incubation, tube formation was examined visually in three random images imported at a magnification of 100× by inverted microscope, and the length and the area of tubular structure in each image (1 × 1 mm) were analyzed using Adobe photoshop CS3 software. Data are shown as mean ± SEM.

##### Histology and PMNs counting

In experiment 1, PMNs were detected using paraffin sections stained with periodic acid - Schiff's (PAS) reagent for 10 min, and then counterstained with hematoxylin (Certistain, Merck, Germany) [30]. This PAS stain was used on each tissue block, and 5 fields per section were examined at × 400 magnification. Although red blood cells and other immune cells, such as macrophages are stained in the CL tissue, therefore in addition to positive cells by PAS staining, we checked the shape of nuclear of cells and assessed segmented granulocytes as PMNs. Thus, we could distinguish PMNs especially neutrophils from other PAS stained positive cells, such as red blood cells and macrophages. Quantification of the number of PMNs was performed independently by 3 observers. The numbers of PMNs/0.04 mm^2 ^(200 × 200 μm) were independently counted under a light microscope. Intra- and inter-observer differences were < 10%. Data are shown as mean ± SEM.

### RNA isolation and real-time PCR

Total RNA was extracted following the protocol of Chomczynski and Sacchi using TRIzol reagent [31] as in our previous study [32]. The extracted total RNA was stored in RNA storage solution (Ambion, Texas, USA) at -80°C until use for cDNA production. RNA samples were treated with DNase using the RQ1 RNase-Free DNase kit (Promega. Co., Madison, WI, USA) as in our previous study [32]. The synthesized cDNA was stored at -30°C.

PCRs were quantified using a LightCycler (Roche Diagnostics, Indianapolis, IN, USA) and a commercial kit (LightCycler FastStart DNA Master SYBR Green I, Roche Diagnostics) as describe previously [32]. The quantification of mRNA expression was performed using Light Cycler Software (Version 3.5; Roche Diagnostics). The primers used for real-time PCR were as follows: IL-8 (NM_173925, 170 bp), forward, 5- CCTCTTGTTCAATATGACTTCCA-3 and reverse, 5- GGCCCACTCTCAATAACTCTC-3; GAPDH (NM_001034034, 160 bp), forward, 5-CTCTCAAGGGCATTCTAGGC-3and reverse, 5-TGACAAAGTGGTCGTTGAGG-3. The amplification program consisted of 15 min activation at 95°C followed by 40 cycles of PCR steps (15 sec denaturation at 94°C, 30 sec annealing at 58°C and a 20 sec extension at 72°C). The quantification of mRNA expression was done using Light Cycler Software (Version 3.5; Roche). The values were normalized using GAPDH as the internal standard. Following electrophoresis of the PCR, the target band was cut out, PCR amplicons were sequenced in both 5' and 3' orientation to confirm the PCR amplicons as IL-8 and GAPDH using an Applied Biosystems 3730 × l DNA Analyzer (Applied Biosystems, Foster City CA, USA).

### Statistical analysis

All data are presented as means ± SEM. The statistical significance of differences about PMNs number, IL-8 expression of both mRNA and protein, PMN migration levels, LEC proliferation and LEC matrigel assay was assessed by one-way ANOVA followed by Bonferroni's multiple comparison test. Probabilities less than 5% (P < 0.05) were considered significant.

## Results

### Quantification of PMNs and IL-8 mRNA expression and protein in the CL during the estrous cycle

**Figure 1:**
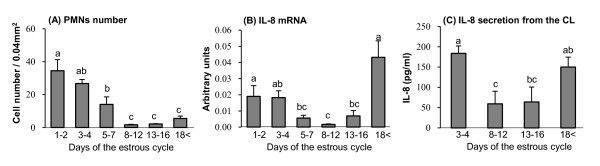
**Number of PMNs, IL-8 mRNA expression, IL-8 secretion in the bovine CL throughout the luteal phase**. Figure 1A, B and C indicate the number of PMNs, IL-8 mRNA expression in the CL and IL-8 concentration within the supernatant of the CL tissue during the estrous cycle, respectively. Values are shown as the mean ± SEM. Different superscript letters indicate significant differences (P < 0.05) as determined by ANOVA followed by the Bonferroni's multiple comparison test.

**Figure 2:**
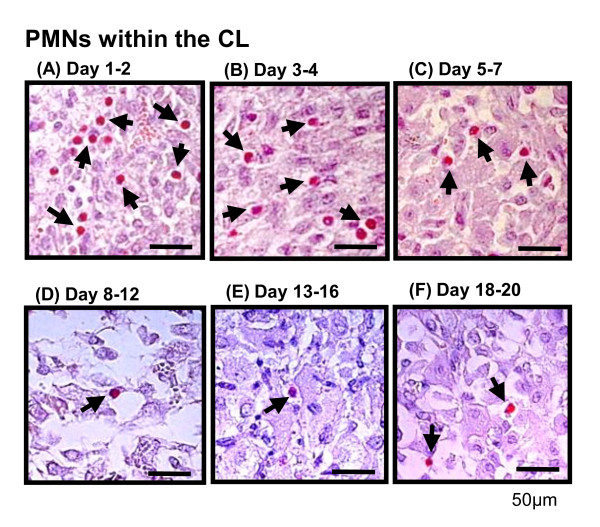
**Distribution of PMNs in the bovine CL throughout the luteal phase**. Figure 2A-F indicate typical images of PMNs by PAS staining in the CL during the estrous cycle. Black arrows indicate PMNs. PMNs were clear granule and segmented nuclear. As Figure 1A, on day 1-2 and 3-4 of the estrous cycle, high numbers of PMNs were detected within the luteal tissue (Figure 2A and B). Scale bars indicate 50 μm.

The number of PMNs, IL-8 mRNA and protein during the estrous cycle are shown in Figure 1A-C. PMNs within the CL were detected (red-stained cells indicating arrows) using paraffin sections stained with periodic acid - Schiff's (PAS) reagent (Figure 2A-F). On day 1-2 of the estrous cycle, high numbers of PMNs were detected within the luteal tissue (Figure 1A and 2A). Continuously, PMNs were present in substantially higher numbers on Day 3-4 and 5-7 in the early stage of the estrous cycle (Figure 1A, 2B and 2C, P < 0.01), and then reduced mid- (Figure 1A and 2D), late- (Figure 1A and 2E) and luteal regression (Figure 1A and 2F).

**Figure 3:**
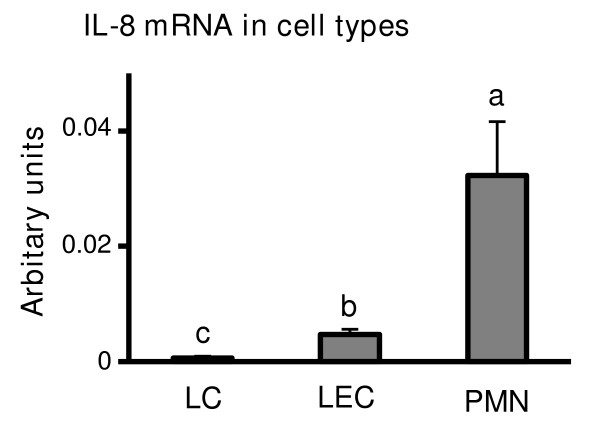
**IL-8 mRNA expression in LCs, LECs and PMNs**. Figure 3 show IL-8 mRNA expression depending on the types of cells, such as LCs, LECs and PMNs. Values are shown as the mean ± SEM. Different superscript letters indicate significant differences (P < 0.05) as determined by ANOVA followed by the Bonferroni's multiple comparison test.

IL-8 mRNA expression was higher in the early (especially Days 1-4) and regression luteal phases (days 18 or greater) compared with mid and/or late luteal phases of the estrous cycle (Figure 1B). IL-8 concentration within the supernatant of the CL tissue is shown in Figure 1C. Early CL had highest levels of IL-8 during the estrous cycle, and IL-8 in regressing CL was higher than in mid and late CL (Figure 1C). Also, we investigated IL-8 mRNA expression depending on the LCs, LECs and PMNs (Figure 3). PMNs were the highest expression of IL-8 mRNA, and IL-8 mRNA was expressed higher levels in LECs compared to LCs.

### PMN migration by CL supernatant derived from the early and mid CL, and PMN migration by the early-phase CL supernatant with or without anti-IL-8 antibody pre-treatment

**Figure 4:**
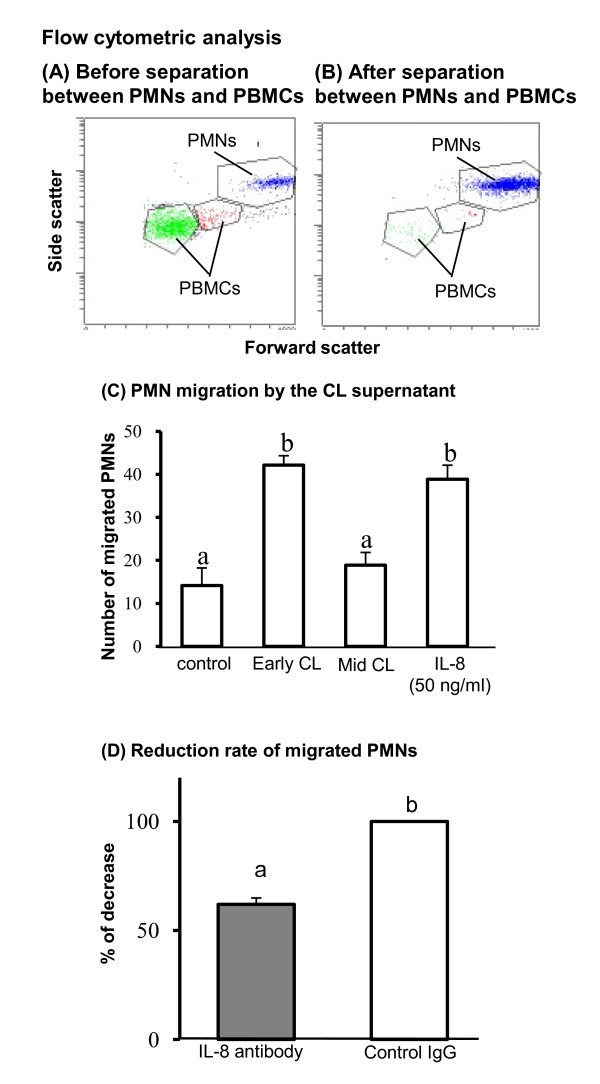
**PMN migration by the supernatant of early and mid CL culture and PMN migration levels with or without anti-IL-8 Ab pre-treatment**. Before PMNs migration experiment, we evaluated the purity of PMNs separation from white blood cells using flow cytometry. Figure 4A indicates the plotting data of peripheral white blood cells (not separate between PMNs and pheripheral blood mononuclear cells (PBMCs)). After removing PBMCs layer using Lymphoprep, we observed clear separation of PMN population (Figure 4B) and the purity of PMNs was > 95% and these cells resulted in nearly pure granulocyte populations as determined by flow cytometric evaluation. PMN migration by the supernatant obtained from early- and mid-phase CL cultures are shown in Figure 4C. The supernatant of the early CL significantly induced PMN migration as well as IL-8 (50 ng/ml as a positive control) compared with control. Figure 4D indicates the reduction ratio of migrated PMNs using the supernatant of the early CL with anti-IL-8 monoclonal antibody or control IgG. All values are the mean ± SEM. Different superscript letters indicate significant differences (P < 0.05) as determined by ANOVA followed by the Bonferroni's multiple comparison test.

Based on the results of Figure 1-2, we hypothesized that PMN infiltration is dependent on a chemoattractant produced by the CL, especially IL-8. Before PMNs migration experiment, we evaluated the purity of PMNs separation from white blood cells using flow cytometry. Figure 4A indicates the plotting data of peripheral white blood cells (not separate between PMNs and pheripheral blood mononuclear cells (PBMCs)). After removing PBMCs layer using Lymphoprep, we observed clear separation of PMN population (Figure 4B) and the purity of PMNs was > 95% and these cells resulted in nearly pure granulocyte populations as determined by flow cytometric evaluation. PMN migration by the supernatant obtained from early- and mid-phase CL cultures are shown in Figure 4C. The data show that the supernatant obtained from the early CL significantly induced PMN migration as well as IL-8 (50 ng/ml as a positive control) compared with control (culture medium was applied in the bottom chamber). The supernatant from the mid-phase CL could not induce PMN migration. To investigate the possible role of IL-8 as a neutrophil-specific chemoattractant in early-phase CL tissue culture supernatant, we inhibited IL-8 function using anti-IL-8 monoclonal antibody react with the bovine IL-8 (Ab; MAB1044, Millipore Corporation, Australia) and then performed a transmigration assay. Based on the PMN migration ratio in the without anti-IL-8 antibody pre-treatment group (control IgG treatment), the level of PMN migration was significantly lower in the IL-8 antibody pre-treatment group compared with the control IgG-treatment group (n = 4, Figure 4D).

### Effect of IL-8 and PMNs on the function of luteal endothelial cells

**Figure 5:**
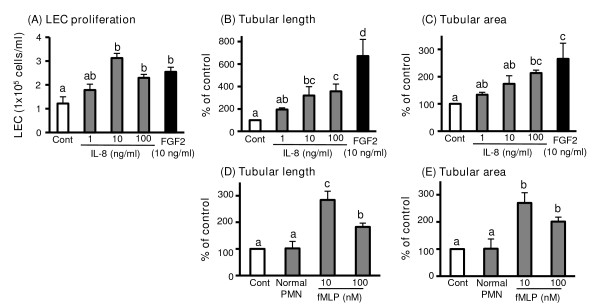
**Effects of IL-8 and activated PMNs on live cell number and tubular formation of LEC**. Figure 5A-C shows the effect of IL-8 and PMNs on the function of LECs *in vitro*. Figure 5A shows that IL-8 at 10 and 100 ng/ml as well as FGF2 stimulated live cell number of LECs. The data show that IL-8 at 10 and 100 ng/ml as well as FGF2 stimulated the length of capillary-like tube (Figure 5B), and the area of tubular structure (Figure 5C, n = 4). Figure 5D and E shows the effects of PMNs on tubular formation of LECs. PMNs from the early luteal phase were cultured to activate in the presence of fMLP (10 and 100 nM) or without fMLP. An in vitro matrigel assay, the supernatant of activated-PMNs stimulated sprouting of capillary-like structures of LECs (n = 4, P < 0.05) compared with control and normal PMNs (non-activated supernatant of PMNs). Values are shown as the mean ± SEM. Different superscript letters indicate significant differences (P < 0.05) as determined by ANOVA followed by the Bonferroni's multiple comparison test.

**Figure 6:**
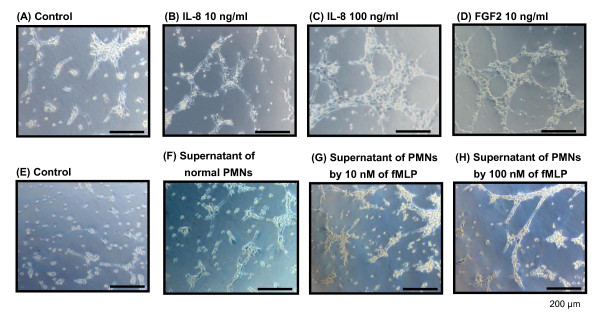
**Image of tubular formation of LEC by IL-8 and activated PMNs**. Typical images of tubular formation of LEC of control, IL-8 (10 and 100 ng/ml), FGF2 (positive control), fMLP-stimulated PMNs (10 and 100 nM) and normal PMNs are shown in Figure 6A-H, respectively. Scale bars indicate 200 μm

Figure 5A-E shows the effect of IL-8 and PMNs on the function of luteal endothelial cells (LEC) *in vitro*. FGF2 (10 ng/ml) was used as a positive control as general angiogenic factor. In Figure 5A, the data show that IL-8 at 10 and 100 ng/ml as well as FGF2 stimulated live cell number of LECs at 48 h compared with control. To investigate the ability of IL-8 and PMNs for angiogenesis, LEC capillary tube formation was evaluated using matrigel and tubular formation was assessed by the length of capillary-like tube structure and the area of tubular structure as previously described [29]. The data show that IL-8 at 10 and 100 ng/ml as well as FGF2 stimulated the length of capillary-like tube structure (Figure 5B) and the area of tubular structure (Figure 5C) at 8 h compared with control (n = 4, P < 0.05). Typical images of tubular formation of LEC of control, IL-8 (10 and 100 ng/ml) and FGF2 are shown in Figure 6A-D, respectively.

To investigate of PMNs for the function of LEC, PMNs from the early luteal phase were cultured to activate in the presence of N-formyl-methionyl-leucyl-phenylalanine (fMLP; 10 and 100 nM) or without fMLP as control for 1 h at 37°C as previously described [15]. After stimulation of PMNs by fMLP, supernatant of activated PMNs was harvested and then used for the assessment of capillary tube formation (Figure 5D and 5E). An in vitro matrigel assay, the supernatant of activated-PMNs stimulated sprouting of capillary-like structures of LECs (n = 4, P < 0.05) compared with control (culture medium alone) and normal PMNs (un-activated supernatant of PMNs). Typical images of tubular formation of LEC of control, fMLP-stimulated PMNs (10 and 100 nM) and normal PMNs are shown in Figure 6E-H, respectively.

## Discussion

This study demonstrated the presence of PMN populations and IL-8 expression (mRNA and protein) within the CL during the estrous cycle in cows. Particularly, PMNs and IL-8 expression were predominant in the very early stage of the luteal phase. The early bovine CL induced PMN migration at least in part *via *IL-8 *in vitro*, and PMNs as well as IL-8 stimulated capillary-like structures of LECs *in vitro*. These findings suggest that PMNs and IL-8, a neutrophil-specific chemoattractant, have important roles in CL angiogenesis in cows.

The CL formation occurs by the rapidly induced terminal differentiation of granulosa and theca cells into luteal cells and promotes tissue transformation including break-down of the follicular basal lamina and migration of various cell types, such as leukocytes and endothelial cells into the granulosa layer, which eventually leads to neovascularization. In the present study, the early bovine CL, specifically days 1-4 of the estrous cycle possessed a large number of PMNs together with high levels of IL-8 expression (Figure 1). Therefore, we hypothesized that PMNs infiltrated *via *IL-8 in the developing CL may play a role in angiogenesis of the CL.

To investigate the role of PMNs and IL-8 in CL development, we focused on the mechanism of PMN migration in the bovine CL. In the present study, we investigated the factors secreted from the CL to induce migration of PMNs. The early CL produced a greater quantity of IL-8, a good candidate of neutrophil-specific chemoattractant compared to the mid CL. Indeed, PMN migration was stimulated by the supernatant from the early CL but not from the mid CL, and this active PMN migration in the early CL was partially inhibited by pre-treatment with anti-IL-8 antibody (Figure 4D). However, treatment of IL-8 antibody could not completely inhibited (only 40%) the PMN migration by the supernatant of the early CL. Interestingly, as in the case of IL-8, FGF2 and VEGFA (10 and 100 ng/ml) stimulated PMN migration *in vitro *(our preliminary study, data not shown), and FGF2 and VEGFA are also expressed at high levels within the developing CL. Indeed, Ancelin et al. demonstrated that VEGFA was chemotactic for neutrophils in humans and that a neutralizing with anti-VEGF antibody blocked this effect [14]. FGF2 also enhanced recruitment of neutrophils in rats [33]. Therefore, we speculate that IL-8, VEGFA, and FGF2 are acting synergistically as stimulators of PMN recruitment in the early CL in cows. We have to continuously investigate about this hypothesis in future study.

To elucidate our hypothesis that PMNs and IL-8 may play a role in angiogenesis of the CL, we investigated the effects of PMNs and IL-8 on the function of LECs *in vitro*. Similar to FGF2 as a strong angiogenic factor, IL-8 increased proliferation of LECs and stimulated sprouting of capillary-like structures of LECs in *in vitro *matrigel assay. On the other hand, it has been reported that human PMNs stimulated by 100 nM fMLP secretes substantial amount of IL-8 at 1 h after stimulation and is able to induce sprouting of capillary-like structures [15,29]. As referred these previous study, the supernatant of activated-PMNs by fMLP as well as FGF2 in the present study, stimulated sprouting of capillary-like structures of LECs in *in vitro *matrigel assay, suggesting the crucial role of PMNs-IL-8 axis in the developing CL in cows. Actually, PMNs and IL-8 can induce angiogenesis *in vivo *[16,21] and *in vitro *[15,29], suggesting that the PMNs-IL-8 duo may function not only in the induction of tissue inflammation and wound healing, but also in the regulation of angiogenesis.

Angiogenesis is a critical component of normal luteal function, and FGF2 and VEGFA are major angiogenic factors in the bovine CL [34-37]. Indeed, Robinson et al., demonstrated that in a luteal angiogenesis culture system (involves luteal cells, endothelial cells and smooth muscle cells), a physiological dose (1 ng/ml) of FGF2 and VEGFA stimulates the degree of the endothelial cell network [35]. In addition, we recently showed that treatment with FGF2 and VEGFA antibodies markedly suppressed angiogenesis in the early luteal phase of the bovine CL *in vivo *[37]. VEGFA stimulated the mRNA expression of IL-8 in bovine theca [38] and human endothelial cells [15]. These findings suggest that PMNs and IL-8 together with FGF2 and VEGFA actively contribute to induce angiogenesis during the early CL development.

Similarly to PMNs in the present study, macrophages and lymphocytes have been identified in the developing CL in several species [11,39-41], and have been suggested to be putative intraovarian regulators. Indeed, peripheral blood lymphocytes and macrophages indicated a luteotrophic effect as these cells stimulated P secretion upon co-culture with granulosa cells [42-44]. Meanwhile, Furukawa et al. revealed that platelets are novel regulators of neovascularization and luteinization during the early luteal phase in humans [45]. An increased number of platelets localized to extravascular sites among luteinizing granulosa cells after ovulation and gradually decreased toward the mid luteal phase [45], that are similar to the PMN profile observed in the present study. Moreover, platelets stimulated P secretion in luteinizing granulosa cells and endothelial cell migration [45]. Taken together, these findings suggest that immune and blood cells are active components in CL development including angiogenesis, luteinization and P secretion.

## Conclusion

This study showed that PMNs migrate into the early CL partially due to its major chemoattractant IL-8 produced at high levels in the CL, and they are most likely to be involved in the regulation of angiogenesis. Thus, PMNs are novel regulators of early CL development in the cow.

## Competing interests

The authors declare that they have no competing interests.

## Authors' contributions

SJ participated carried out all experiments and drafted the manuscript. KS participated in the design of the study, experiments of *in vitro *angiogenesis and drafted the manuscript. AN and AK collected the materials, and helped to immunohistochemistry and *in vitro *cell culture experiments. HJS and TS helped the design of the study and instructed technique of the experiments. AM supervised the study and helped to draft the manuscript. All authors read and approved the final manuscript.
